# Daily emergency department volume and in-hospital mortality: the importance of temporal alignment

**DOI:** 10.1186/s13049-026-01703-4

**Published:** 2026-09-23

**Authors:** Mert Gültekin

**Affiliations:** Department of Emergency Medicine, Şanlıurfa Training and Research Hospital, Şanlıurfa, 63200 Türkiye

Dear Editor, 

I read with great interest the multicentre study by Raven et al. examining annual emergency department (ED) volume and relative daily volume in relation to in-hospital mortality across common presenting complaints [1]. The distinction between structural scale and short-term variation in attendance is clinically important, and the finding that excess mortality in the Dutch cohort was concentrated on low-volume rather than high-volume days is particularly thought-provoking. I would like to raise a methodological issue concerning whether whole-day attendance is temporally aligned with the individual patient whose outcome is being modelled.

In the daily-volume analysis, all ED visits occurring on a calendar day contributed to that day’s volume, which was standardised against the usual daily volume of the same ED [1]. Consequently, patients presenting early and late on the same day receive the same volume exposure. For a patient who arrives in the morning and completes assessment, treatment, or disposition before the evening, however, visits occurring later that day could not have influenced the operational conditions experienced during that patient’s care. Whole-day attendance may therefore be a useful descriptor of an ED-day while remaining an imperfect patient-level measure of short-term workload.

This distinction is not merely theoretical. Abir et al. measured occupancy during the hour of the disposition decision and explicitly noted that daily-level measurement may mask substantial within-day variation; their example similarly emphasises that a patient managed early in the day cannot be affected by crowding that develops later [2]. In a Dutch prospective study, van der Veen et al. recorded the number of patients physically present in the ED at the time of the index patient’s registration, thereby anchoring an operational measure to the encounter itself [3]. Real-time crowding research has likewise evaluated occupancy and workload measures at short intervals rather than assigning a single value to an entire day [4]. These approaches illustrate a broader point: when a system state is proposed as an exposure for an individual outcome, the system state is most informative when it corresponds to the period during which the patient was actually exposed to it.

The sensitivity analyses by Raven et al. appropriately adjusted for time of day, ambulance transport, day of week, and month of presentation [1]. These adjustments strengthen control of confounding but do not change the temporal construction of the daily-volume variable. Adding arrival time as a covariate is different from redefining workload so that it only incorporates operational conditions preceding or overlapping the patient’s care.

A second issue follows from the level at which relative daily volume varies. Daily volume is an ED-day-level characteristic, whereas mortality is analysed at the individual-visit level. Patients treated in the same ED on the same day therefore share the same principal exposure and may also share unmeasured day-specific conditions, such as staffing, bed availability, downstream capacity, or operational disruption. The reported mixed-effects models include ED location as a random intercept [1], but it is not clear whether dependence among patients within the same ED-day was additionally accounted for. Clarifying this point, or repeating the analysis with ED-day clustered standard errors or a nested ED-day effect, could help determine the precision of the daily-volume estimates.

The Dutch organisational context provides a further reason not to equate observed attendance with experienced operational pressure. Raven et al. note that regional coordination and temporary ambulance diversion are available in many Dutch regions [1]. Ambulance diversion has also been used as an indicator of ED crowding in real-time operational research [4]. Thus, on at least some days, observed attendance may partly reflect a system response to capacity constraints rather than underlying demand alone. More broadly, established models conceptualise ED crowding as the interaction of input, throughput, and output rather than patient arrivals alone [5], and hospital bed occupancy can influence ED length of stay and admission behaviour even when mortality is unchanged [6]. These observations offer plausible alternatives to interpreting low attendance as a genuinely low-workload state.

A useful next analysis would therefore anchor workload to the patient’s care interval—for example, arrivals during a prespecified period before presentation, contemporaneous ED census or occupancy, or the number of patients whose ED stays overlap the index patient’s stay. Where available, periods of ambulance diversion or other capacity-management responses could be examined separately. If the excess mortality on low-volume days persisted after such temporally aligned analyses and ED-day clustering, the finding would provide stronger evidence that unusually low operational activity identifies a clinically meaningful system state. Conversely, attenuation would suggest that whole-day attendance is functioning partly as a proxy for other time-varying operational conditions.

Raven et al. provide valuable evidence that structural ED volume and short-term variation in activity should not be treated as interchangeable. Aligning the measurement of daily workload with the individual patient’s actual period of care may help clarify the intriguing association between low-volume days and mortality.

## Data Availability

No datasets were generated or analysed during the current study.
